# Metatranscriptomics Supports the Mechanism for Biocathode Electroautotrophy by “*Candidatus* Tenderia electrophaga”

**DOI:** 10.1128/mSystems.00002-17

**Published:** 2017-03-28

**Authors:** Brian J. Eddie, Zheng Wang, W. Judson Hervey, Dagmar H. Leary, Anthony P. Malanoski, Leonard M. Tender, Baochuan Lin, Sarah M. Strycharz-Glaven

**Affiliations:** United States Naval Research Laboratory, Washington, DC, USA; University of Ghent

**Keywords:** biocathode, electroautotroph, metatranscriptomics, microbial electrochemical system

## Abstract

Biocathodes provide a stable electron source to drive reduction reactions in electrotrophic microbial electrochemical systems. Electroautotrophic biocathode communities may be more robust than monocultures in environmentally relevant settings, but some members are not easily cultivated outside the electrode environment. We previously used metagenomics and metaproteomics to propose a pathway for coupling extracellular electron transfer (EET) to carbon fixation in “*Candidatus* Tenderia electrophaga,” an uncultivated but dominant member of an electroautotrophic biocathode community. Here we validate and refine this proposed pathway using metatranscriptomics of replicate aerobic biocathodes poised at the growth potential level of 310 mV and the suboptimal 470 mV (versus the standard hydrogen electrode). At both potentials, transcripts were more abundant from “*Ca.* Tenderia electrophaga” than from any other constituent, and its relative activity was positively correlated with current. Several genes encoding key components of the proposed “*Ca.* Tenderia electrophaga” EET pathway were more highly expressed at 470 mV, consistent with a need for cells to acquire more electrons to obtain the same amount of energy as at 310 mV. These included *cyc2*, encoding a homolog of a protein known to be involved in iron oxidation. Mean expression of all CO_2_ fixation-related genes is 0.27 log_2_-fold higher at 310 mV, indicating that reduced energy availability at 470 mV decreased CO_2_ fixation. Our results substantiate the claim that “*Ca.* Tenderia electrophaga” is the key electroautotroph, which will help guide further development of this community for microbial electrosynthesis.

**IMPORTANCE** Bacteria that directly use electrodes as metabolic electron donors (biocathodes) have been proposed for applications ranging from microbial electrosynthesis to advanced bioelectronics for cellular communication with machines. However, just as we understand very little about oxidation of analogous natural insoluble electron donors, such as iron oxide, the organisms and extracellular electron transfer (EET) pathways underlying the electrode-cell direct electron transfer processes are almost completely unknown. Biocathodes are a stable biofilm cultivation platform to interrogate both the rate and mechanism of EET using electrochemistry and to study the electroautotrophic organisms that catalyze these reactions. Here we provide new evidence supporting the hypothesis that the uncultured bacterium “*Candidatus* Tenderia electrophaga” directly couples extracellular electron transfer to CO_2_ fixation. Our results provide insight into developing biocathode technology, such as microbial electrosynthesis, as well as advancing our understanding of chemolithoautotrophy.

## Abstract (precis)

Bacteria that directly use electrodes as metabolic electron donors (biocathodes) have been proposed for applications ranging from microbial electrosynthesis to advanced bioelectronics for cellular communication with machines. However, just as we understand very little about oxidation of analogous natural insoluble electron donors, such as iron oxide, the organisms and extracellular electron transfer (EET) pathways underlying the electrode-cell direct electron transfer processes are almost completely unknown. Biocathodes are a stable biofilm cultivation platform to interrogate both the rate and mechanism of EET using electrochemistry and to study the electroautotrophic organisms that catalyze these reactions. Here we provide new evidence supporting the hypothesis that the uncultured bacterium “*Candidatus* Tenderia electrophaga” directly couples extracellular electron transfer to CO_2_ fixation. Our results provide insight into developing biocathode technology, such as microbial electrosynthesis, as well as advancing our understanding of chemolithoautotrophy.

## INTRODUCTION

Biocathodes are components of some bioelectrochemical systems (BES) in which microbial electrode catalysts use the electrode as an electron donor to drive cellular metabolism. Over the last decade, biocathodes have been explored for improving energy recovery in microbial fuel cells (MFCs) and electrode-driven bioremediation and, more recently, to produce chemicals in a process known as microbial electrosynthesis (reviewed in references 1 and 2). Despite widespread interest in biocathodes for biotechnology applications, little is understood about the underlying mechanisms of extracellular electron transfer (EET) for biocathode microorganisms. Although biocathode EET has been demonstrated for a variety of microorganisms, including acetogens (3) and a methanogenic archaeon (4), mechanistic studies aimed at identifying EET conduits from the electrode to cells are limited (5–7).

The *Marinobacter-Chromatiaceae-Labrenzia* (MCL) biocathode is a self-regenerating, self-sustaining, microbial community and has served in our laboratory as a model system for the exploration of aerobic electroautotrophic microbial communities using an omics approach (8, 9). MCL forms a heterogeneously distributed biofilm with cellular aggregates up to 20 µm thick (9) and reproducible electrochemical features following inoculation of a portion of the biofilm into a new reactor. It is proposed that MCL reduces O_2_ with electrons supplied solely by the cathode, directing a portion of the acquired energy and electrons for autotrophy. Cyclic voltammetry (CV) revealed a sigmoid-shaped dependency for current associated with catalytic O_2_ reduction (turnover current) dependent upon electrode potential (10). It is proposed that this dependency reflects Nernstian behavior of the heterogeneous electron transfer reaction (across the biofilm/electrode interface) that is mediated by a redox cofactor, which is fast, reversible, and not the rate-limiting step in direct electron uptake from the cathode by the biofilm (9, 11, 12). Electroautotrophic growth is assumed for MCL based on an increase in biomass correlated to increasing current, a lack of organic carbon in the bioelectrochemical reactor, and identification of an active Calvin-Benson-Bassham (CBB) cycle (13). Electroautotrophic growth of isolates at potentials greater than −100 mV versus the standard hydrogen electrode (SHE) has thus far been demonstrated only for the aerobic Fe(II)-oxidizing bacteria *Mariprofundus ferrooxydans* PV-1 and *Acidithiobacillus ferrooxidans* (14, 15), while other autotrophs require supplemental energy from light or hydrogen for initial growth on a cathode (6, 16). However, recent reports indicate that communities enriched on high potential O_2_-reducing biocathodes reproducibly contain large populations of *Gammaproteobacteria* (17, 18).

Previous metagenomic and proteomics studies of MCL have led to the identification of putative EET and CO_2_ fixation mechanisms (13, 19), although efforts at cultivation have not yielded the proposed electroautotroph, “*Candidatus* Tenderia electrophaga” (20). Other work has resulted in isolates from aerobic biocathode enrichments, but these have not been autotrophic (21, 22). Metaproteomic analysis suggested that proteins that may be involved in EET, including a homolog of Cyc2, a cytochrome known to be involved in Fe(II) oxidation in *A. ferrooxidans* (13), are present at high levels in the biofilm. Subsequent metaproteomic analysis of the biofilm at two different electrode potentials showed that some components of the electron transport chain (ETC) are differentially expressed, including an ortholog of Cyc1, thought to be involved in Fe(II) oxidation in *M. ferrooxydans* PV-1 (23), and a hypothetical protein homologous to the terminal oxidase cytochrome cbb_3_ subunit CcoO (19). However, methodological limitations meant that the nine proteins that were detected significantly more often at one potential than at another were likely to represent a small fraction of the response to changing potential in “*Ca.* Tenderia electrophaga” (19). More-precise quantification of the changes in relative gene expression obtained using RNA sequencing (RNA-seq) (24) can be used to understand the molecular mechanisms used for growth on the cathode.

Building upon our previous work (9, 13, 19), we applied RNA-seq to MCL to compare the levels of gene expression of proposed EET and CO_2_ fixation pathways for “*Ca.* Tenderia electrophaga” at two different potentials. Adjusting the applied potential from 310 mV versus SHE (optimal for growth) to 470 mV (suboptimal for growth) results in a decrease in the ΔG°′ value for the reduction of O_2_ by about one-third. From results of previous metaproteomic experiments run under identical conditions, the change in potential of the electron donor is expected to result in changes in gene expression to compensate for the change in energy availability. Our metatranscriptomic results support previously hypothesized roles of some protein complexes (13, 19, 23) and reveal possible EET roles for other proteins. We also provide further evidence that “*Ca.* Tenderia electrophaga” is the keystone species in this community and is strongly associated with electron uptake rates. The results from the metatranscriptomics analyses provide insight into the molecular mechanisms involved in electroautotrophic growth on a cathode, enabling the development of biocathodes for possible future applications, including synthesis of value-added compounds or biofuels as well as potential ET components for microbial bioelectronics.

## RESULTS

### Biocathode-MCL metatranscriptome.

Eight BES were grown under previously described standard conditions at an applied electrode potential of 310 mV SHE. Seven of the eight biological replicates reached the maximum amplitude of current density within 5 days (Table 1; see also Fig. S1 in the supplemental material). Replicate S2A took 2 days longer. The midpoint potential (E_M_) of each BES measured by CV once steady-state current was achieved was similar to that previously reported (9, 10, 13, 19) at ca. 440 mV versus SHE (Table 1). Following CV, the electrode potential was either returned to 310 mV or adjusted to 470 mV, a potential at which we expect the biofilm to recover less energy per electron based on previous experiments. After 52 h, the reactors were disconnected and biofilm samples were scraped off the electrodes and preserved in RNAlater within the span of 2 min. RNA was extracted from all eight samples on the same day to reduce batch effects.

10.1128/mSystems.00002-17.1 FIG S1  Chromoamperometry of biofilms grown at 310 mV and either switched to 470 mV or maintained at the optimal potential. (A) Chronoamperometry of inoculum 1. (B) Chronoamperometry of inoculum 2. Vertical lines indicate the times at which the CV was measured and the potential was switched. Sample ID string: O, optimal (310 mV); S, suboptimal (470 mV); 1 or 2, inoculum 1 or 2, respectively; A or B, replicate A or B, respectively. Download FIG S1, TIF file, 0.5 MB. Copyright © 2017 Eddie et al. 2017 Eddie et al. This content is distributed under the terms of the Creative Commons Attribution 4.0 International license.

**TABLE 1:** Sample and sequencing summary

Sample ID^a^	Inoculum	Potential (mV vs SHE) at sampling	Hours to maximum current	Maximum current (µA cm^−2^)	Current just before CV (µA cm^−2^)	Current at sampling (µA cm^−2^)	E_M_ (mV vs SHE)	No. of reads that passed QC^b^	% reads unambiguously aligned
O1A	1	310	109	−50.2	−20.5	−35.3	440	16,168,243	37.66
O1B	1	310	116	−31.9	−23.2	−29.3	460	14,694,809	84.58
O2A	2	310	109	−14.3	−14.5	−12.6	480	4,498,496	62.64
O2B	2	310	85	−13.4	−8.2	−8.44	500	9,957,581	71.48
S1A	1	470	104	−54.3	−22.7	−7.29	420	13,782,405	67.30
S1B	1	470	109	−64.1	−34.9	−23.6	450	9,855,410	83.65
S2A	2	470	161	−53.5	−53.4	−52.2	430	15,415,038	90.03
S2B	2	470	90	−14.9	−14.5	−19.6	454	13,921,246	82.49

a Sample identifier (ID) string: O, optimal (310 mV); S, suboptimal (470 mV); 1 or 2, inoculum 1 or 2, respectively; A or B, replicate A or B, respectively.

b QC, quality control.

**FIG 1:**
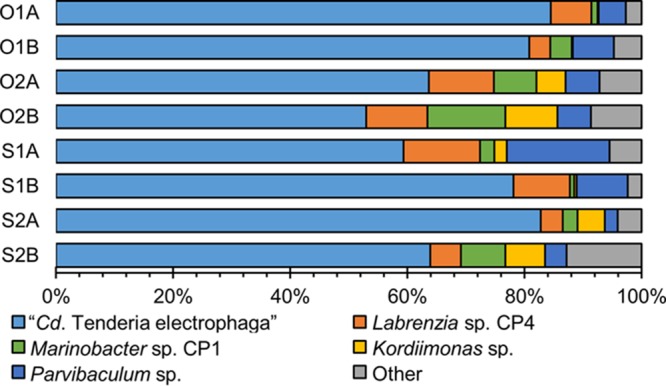
Relative activity of the five major constituents of biocathode-MCL. The proportion of reads matching a genome or bin normalized by the length of the genome or bin was taken as a proxy for activity. Sample labels are as described for Table 1.

MiSeq mRNA sequencing yielded between 4.5 and 16.2 million reads per replicate sample that passed quality control steps, and the data were used for read alignment to the metagenome, of which 37.7 to 90.0% could be unambiguously aligned (Table 1). The proportion of reads mapping to a metagenome bin normalized by the relative length of the bin was used as a proxy for the relative activity of MCL constituents. Using this metric, MCL constituents previously implicated in key roles in the biocathode—“*Ca.* Tenderia electrophaga,” *Marinobacter* sp. strain CP1, and *Labrenzia* sp. strain CP4—were highly active in all samples at both potentials (Fig. 1). Ten other metagenome bins were active in all eight samples using a cutoff of 0.01% of relative activity. Two of these, *Parvibaculum* sp. (6.9% mean relative activity) and *Kordiimonas* sp. (3.5% mean relative activity), had activity similar to that of *Labrenzia* sp. strain CP4 (7.9% mean relative activity) and *Marinobacter* sp. strain CP1 (4.8% mean relative activity), indicating that they may also have significant roles in the biocathode community. “*Ca.* Tenderia electrophaga” was the most active constituent in all eight samples, comprising 53% to 84.3% of activity (Fig. 1). *Marinobacter* sp., *Labrenzia* sp., *Kordiimonas* sp., and *Parvibaculum* sp. averaged 24.6% of the remaining activity. These five constituents make up the core of the biofilm community, on average accounting for 93.6% of the community activity. Two-tailed Student’s *t* tests indicated that no constituent had a significant change in activity between the two potentials tested (*P* > 0.09 for all bins).

### Correlation of “*Ca.* Tenderia electrophaga” transcriptional activity to current density.

**FIG 2:**
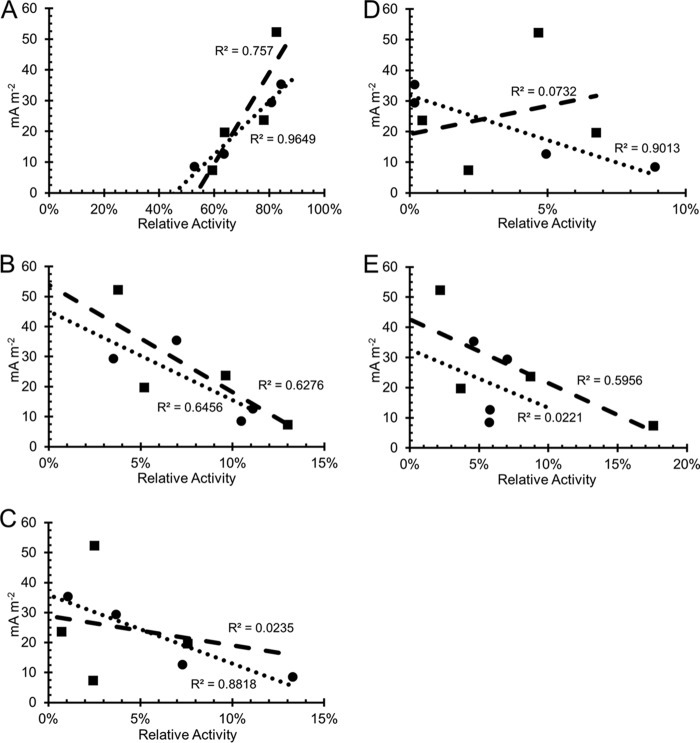
Activity of top five biocathode-MCL constituents versus current density at the optimal and suboptimal electrode potentials. (A) “*Ca.* Tenderia electrophaga” has positive correlations with increasing negative current at both the optimal (circles) and suboptimal (squares) potentials. (B to E) Other active members of the community. *Labrenzia* sp. strain CP4 (B), *Marinobacter* sp. strain CP1 (C), *Kordiimonas* sp. (D), and *Parvibaculum* sp. (E) had a negative or insignificant correlation at both potentials. Linear regression lines are shown with *R*^2^ values.

Activity of “*Ca.* Tenderia electrophaga” was strongly correlated to current density at 310 mV (Fig. 2A) with a Pearson correlation coefficient of 0.982 (*P* = 0.018). The correlation coefficient seen at 470 mV was 0.870, which corresponds to a *P* value of 0.13. The activity of the four most active heterotrophic constituents, *Labrenzia* sp., *Marinobacter* sp., *Parvibaculum* sp., and *Kordiimonas* sp., was negatively correlated or not correlated with current density (Fig. 2B to E). Previous metagenomic and metaproteomic data indicated that “*Ca.* Tenderia electrophaga” is likely an autotrophic organism capable of performing direct EET (13, 19), and the correlation of current density to activity supports this theory. For this reason, we concentrated our analysis of metatranscriptomics data on “*Ca.* Tenderia electrophaga” to focus specifically on how direct EET is linked to CO_2_ fixation.

### Effect of electrode potential on electron transport gene expression.

Efforts to cultivate a representative isolate of “*Ca.* Tenderia electrophaga” from MCL have thus far been unsuccessful, suggesting that other community constituents satisfy unknown requirements for its growth. We therefore used metatranscriptomics to obtain higher-resolution analysis of differentially expressed mRNA levels for proteins suspected to be involved in electroautotrophic growth. A total of 240 genes in “*Ca.* Tenderia electrophaga” were significantly more highly expressed at 470 mV, with a false-discovery rate (FDR) of <0.05, while 140 genes were more highly expressed at 310 mV. This allowed us to test the hypothesis that expression of protein-coding genes involved in respiration and energy flux in the primary electroautotroph is directly influenced by the potential of the electrons supplied by the cathode. A complete list of genes that we propose to be associated with these pathways is presented in Table S1 in the supplemental material and discussed below.

**FIG 3:**
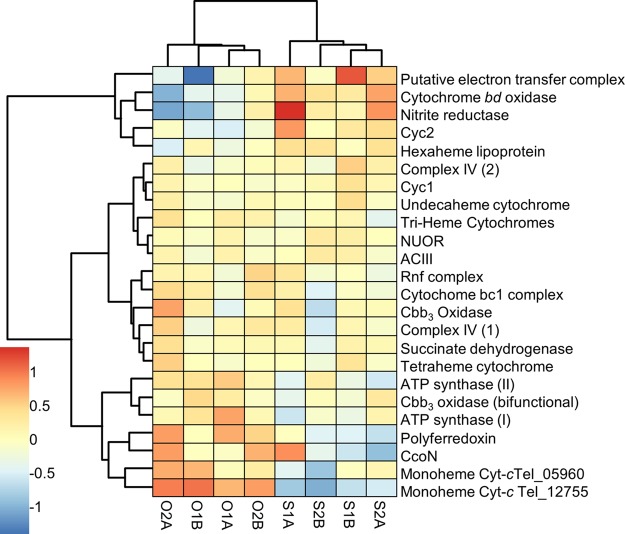
Hierarchical clustering of EET and ETC components by change in expression. Expression was normalized to 0 between biological replicates to focus on changes in expression between 310 mV and 470 mV. Details about genes in each complex can be found in Table S1. Sample identifiers are as described for Table 1.

10.1128/mSystems.00002-17.3 TABLE S1  Expression of ETC components in “*Ca.* Tenderia electrophaga.” Data represent a subset of genes encoding possible ETC or EET components and include complex notation corresponding to the data described for Fig. 3. Data in columns represent a combination of gene annotation, EdgeR output, and calculated relative change in expression. Column heading abbreviations: logFC, log_2_-fold change in expression from 310 mV to 470 mV SHE (genes with a positive value were more highly expressed at 470 mV, and those with a negative value were more highly expressed at 310 mV); FDR, false-discovery rate; CPM, normalized log_2_ counts per million. Coloring of data in the logFC column is as follows: green, value > 0.5; red, value < 0.5. Coloring of data in the FDR column is as follows: green, value < 0.01; yellow, value <0.02; red, value < 0.05. Download TABLE S1, XLSX file, 0.1 MB. Copyright © 2017 Eddie et al. 2017 Eddie et al. This content is distributed under the terms of the Creative Commons Attribution 4.0 International license.

Electrochemical measurements indicate that redox-dependent direct electron transfer occurs between the electrode and “*Ca.* Tenderia electrophaga” (9, 10); therefore, we searched the metatranscriptome for evidence of an EET conduit whose expression might be affected by the change in electrode potential (Table S1). The *cyc*2 homolog in “*Ca.* Tenderia electrophaga” (Tel_03480) was predicted to have a role in EET (13) based on its proposed involvement in iron oxidation and was significantly differentially expressed, with a log_2_-fold change (LogFC) of 0.69 at 470 mV relative to 310 mV. An undecaheme c-cyt (Tel_16545) and a predicted hexaheme lipoprotein (Tel_04230) were previously identified as a possible route(s) for EET in “*Ca.* Tenderia electrophaga” due to the large number of predicted heme binding sites, known to be important for EET in *Shewanella* and *Geobacter* spp., and to conservation among other EET-capable organisms (13). The genes for these proteins were not differentially expressed.

It is thought that soluble, periplasmic cytochromes mediate ET between the outer membrane and the cytoplasmic membrane-bound ETC (25, 26). Several genes previously noted from “*Ca.* Tenderia electrophaga” which may encode proteins involved in transferring electrons across the periplasm to ETC were examined for changes in expression between the two electrode potentials (Table S1). A diheme cytochrome *c*_4_, Cyc1, has been proposed to be a periplasmic electron shuttle in the iron-oxidizing bacterium *M. ferrooxydans* (23). The gene encoding this protein is found in “*Ca.* Tenderia electrophaga,” in a region displaying synteny with two contigs from the *M. ferrooxydans* genome (Fig. S2). It is very highly expressed at both potentials (Table S3) but is not significantly differentially expressed. Another potential periplasmic electron carrier identified from the metatranscriptome that was not previously implicated in MCL EET is a monoheme *c*-type cytochrome (Tel_12755). This gene has the largest change in expression of any annotated cytochrome in “*Ca.* Tenderia electrophaga” (1.62 LogFC more highly expressed at 310 mV), suggesting that it may be important for electron transfer at this lower potential. It is homologous to genes for proteins in several iron-oxidizing bacteria, including *Sideroxydans* (45% amino acid identity), *Gallionella* (42% identity), and *Acidithiobacillus* (42% identity) species. This may make it an important gene to consider in models of iron oxidation.

10.1128/mSystems.00002-17.2 FIG S2  Synteny between “*Ca.* Tenderia electrophaga” (top) and *Mariprofundus ferrooxydans* PV-1 (bottom). The *M. ferrooxydans* genes are found on three contigs in the NCBI database, but two (KR106296.1 and AATS01000009) may be joined, as they have identical sequences at one end. Gray lines indicate regions of homology determined by a tBLASTx search in EasyFig 2.2.2 with E values of less than 1 × 10^−13^ and a minimum length of 30 bp (52). This region has been previously noted by Barco et al. to be syntenous within neutrophilic iron-oxidizing bacteria (23). Download FIG S2, TIF file, 0.9 MB. Copyright © 2017 Eddie et al. 2017 Eddie et al. This content is distributed under the terms of the Creative Commons Attribution 4.0 International license.

Expression was higher for three triheme cytochromes (Tel_16515, Tel_16520, and Tel_16530) at 310 mV, but the results were not statistically significant (*P* > 0.4). Overall, these genes are among those most highly expressed in “*Ca.* Tenderia electrophaga,” indicating their importance for growth at the cathode. They also appear to be cotranscribed with a gene encoding a tetratricopeptide repeat domain (Tel_16525; Table S3), which is known to be involved in protein:protein interaction and may be involved in aligning cytochromes in a bridge configuration as proposed for other EET-capable bacteria (27). Peptides from this tetratricopeptide repeat protein were previously found to be significantly more abundant at the suboptimal potential (19), although in the metatranscriptomic data, this group of four genes was slightly more highly transcribed at 310 mV.

Regardless of the pathway used, the e^−^ need to reach the cytoplasmic membrane-bound ETC, which is the main mechanism for conservation of energy via chemiosmotic gradient formation (28). Metatranscriptomic analysis of the ETC components does not indicate large changes in the expression of most of these genes, which suggests that the same ETC components are used at both electrode potentials. Small (LogFC < 0.4) changes in the ratios of expression for whole pathways may represent subtle adjustments in the ratio of electrons going down each path (Table S1). Whether this is in addition to or instead of the pathway being selected by the potential of the electron donor in a manner reminiscent of the results of recent work on electron acceptors in *Geobacter sulfurreducens* remains to be determined (29, 30).

Most genes encoding components of the ETC were not significantly differentially expressed, although many had small but consistent changes in expression between the two potentials. For example, the genes encoding the alternative complex III (ACIII) and the NADH:ubiquinone oxidoreductase complex (NUOR) were not differentially expressed. However, hierarchical clustering based upon the slight changes of expression among these complexes (Fig. 3) suggests that they are functionally linked. This supports the concept of a role as the reverse electron transport (RET) chain.

Only one operon in “*Ca.* Tenderia electrophaga” contains all four of the canonical genes for a bacterial cytochrome cbb_3_ oxidase (*ccoNOPQ*), but it has relatively low expression under both sets of conditions. However, the genome also encodes several proteins homologous to terminal oxidases (complex IV; Table S1) that could potentially reduce O_2_ and generate proton motive force (PMF). Of these, the genes encoding complex IV-2 had some of the highest total expression of any genes. Furthermore, hierarchical clustering places this complex in a cluster that contains the ACIII and NUOR complexes, along with genes for three groups of potential electron transport proteins or complexes reported above (Cyc1, an undecaheme cytochrome, and three paralogous triheme cytochromes), thus linking EET to generation of membrane potential and the reduction of NAD(P)^+^ for CO_2_ fixation (Fig. 3). The orthologous Cyc1 from *M. ferrooxydans* is thought to be responsible for transferring electrons from the outer membrane to the cytochrome cbb_3_ oxidase (23).

Among the genes significantly more highly expressed at 470 mV, hierarchical clustering reveals a similarity in expression patterns between *cyc2*, a gene encoding a protein that has been proposed to play a role in EET in iron-oxidizing bacteria (31), and a gene for hexaheme lipoprotein previously identified as a potential route for EET (Fig. 3; Table S1) (13). They also have expression similar to that of a cytochrome *bd* oxidase that could accept electrons from the quinone pool and reduce O_2_ and that is significantly more highly expressed at 470 mV. A possible nitrite reductase complex (Tel_15450 to Tel_15465) is also found within this cluster. However, this complex lacks genes encoding proteins that could function as analogs of the membrane-associated proton-pumping CcoN. Thus, it is unlikely to make a substantial contribution to PMF. Genes in another set that are also within this cluster encode a potentially interesting complex: a cupredoxin domain protein, a hypothetical tetraheme protein, and two hypothetical diheme proteins. This group of genes is labeled as a putative electron transfer complex, and the three heme-binding proteins are differentially expressed.

All of the genes encoding components of the two ATP synthase operons were more highly expressed at 310 mV, and five of them have an FDR of <0.05, indicating that the energy levels in “*Ca.* Tenderia electrophaga” are dependent upon the electrode potential (Fig. 3; Table S1). A gene encoding a bifunctional cytochrome cbb3 oxidase, which is a fusion of the catalytic subunit CcoN and the proton-pumping CcoO, displays a similar pattern of expression, and, via hierarchical clustering, these three groups of genes are clustered with several other genes that may be involved in electron transfer. One set of genes encodes a polyferredoxin and three other hypothetical proteins (Tel _00495 to Tel_00510) that are within the syntenous regions described above (Fig. S2). The three genes encoding hypothetical proteins are significantly more highly expressed at 310 mV. We also found genes in this cluster encoding two monoheme soluble *c*-type cytochromes, including Tel_12755, which is significantly more highly expressed at 310 mV.

### Effect of potential on carbon fixation and central carbon metabolism.

**FIG 4:**
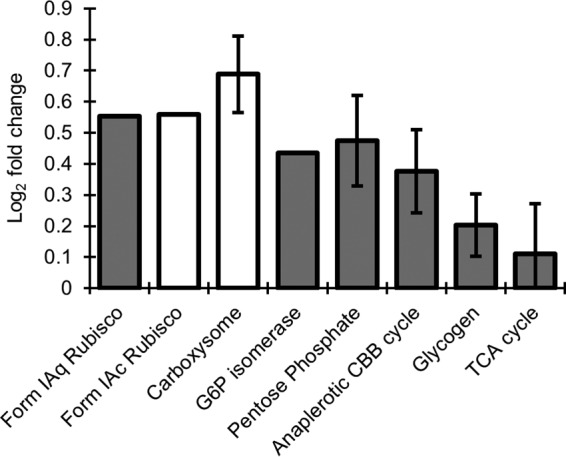
Change in expression of central carbon metabolism genes. Open boxes indicate genes that are more highly expressed at 470 mV, and gray boxes indicate genes that are more highly expressed at 310 mV. Error bars indicate standard deviations of the LogFC of pathways containing three or more genes. Details of the genes included in each category and of their expression can be found in Table S2. Alt. Comp., alternative complex; Cyt., cytochrome; Put., putative; TCA cycle, tricarboxylic acid cycle.

The “*Ca.* Tenderia electrophaga” genome contains all the genes necessary for CO_2_ fixation via the CBB cycle, including two forms of RuBisCO—one associated with carboxysomes (form IAc) and one not associated with carboxysomes (form IAq) (13). The lower energy yield per electron from a cathode poised at 470 mV would result in less energy available for CO_2_ fixation, so the central carbon metabolism of “*Ca.* Tenderia electrophaga” was examined for changes in expression (Fig. 4; Table S3). Genes which could be involved in two or more pathways, for example, the CO_2_ fixation pathway and the pentose phosphate pathway, were separated where possible by examining colocalization and changes in expression among the eight samples. Genes with ambiguous functional roles that could not be assigned to a single pathway were excluded from this analysis. Most genes encoding enzymes of the central carbon metabolism, including the CBB cycle, were more highly expressed at 310 mV, although the absolute magnitude of the difference was relatively low (Fig. 4; Table S3). The small differences in expression of individual genes for the central carbon metabolism were not statistically significant; however, in examining all genes in a given pathway, they were mostly consistent, indicating that the pathways are likely genuinely more highly expressed at 310 mV. The exceptions are the form IAc RuBisCO genes and the carboxysome genes, which were more highly expressed at 470 mV. This suggests a switch to a more efficient enzyme under energy limitation conditions.

10.1128/mSystems.00002-17.4 TABLE S2  Expression of central carbon metabolism genes in “*Ca.* Tenderia electrophaga.” Data represent a subset of genes encoding predicted components of central carbon metabolism and include complex notation corresponding to the data described for Fig. 4. Data in columns represent a combination of gene annotation, EdgeR output, and calculated relative change in expression. Column heading abbreviations: logFC, log_2_-fold change in expression from 310 mV to 470 mV SHE (genes with a positive value were more highly expressed at 470 mV, and those with a negative value were more highly expressed at 310 mV); FDR, false-discovery rate, CPM, normalized log_2_ counts per million. Coloring in logFC column is as follows: green, value > 0.5; red, value < 0.5. Coloring in FDR column is as follows: green, value < 0.01; yellow, value <0.02; red, value < 0.05. Download TABLE S2, XLSX file, 0.04 MB. Copyright © 2017 Eddie et al. 2017 Eddie et al. This content is distributed under the terms of the Creative Commons Attribution 4.0 International license.

## DISCUSSION

Our characterization of the biocathode-MCL community activity by metatranscriptomics supports previous metaproteomic and 16S rRNA amplicon characterizations of biocathode-MCL indicating that there is substantial variability in the abundance of specific bacteria, even between seemingly identical reactors inoculated on the same day with the same inoculum (10, 19). However, the major constituents that are present have been stable for over 6 years, as measured by several methods, including the use of 16S clone libraries, metagenomics, proteomics, 16S amplicon sequencing, and fluorescence *in situ* hybridization microscopy (9, 10, 13, 19, 20), allowing us to use the previously generated metagenome sequencing data to align the metatranscriptomics data. This is important for studies where only limited biomass can be recovered from the electrode surface. As in the previous studies, which examined the abundance of biofilm constituents, changing the electrode potential from 310 mV to 470 mV for 52 h after the community had developed did not result in significant changes in the average relative levels of activity of biocathode constituents between the two potentials. While the inherent variability in relative abundance and activity between replicate reactors makes analysis of the transcriptome data challenging, the differences that we found are more likely to be robust because they are visible above the noise of such large biological variability found by using paired, replicated samples as described by Leary et al. (19).

The magnitude of changes in gene expression was relatively small compared to that seen in other transcriptomic studies (see, e.g., references 32 and 33). However, in one of those studies, replicate samples were acquired by repeatedly sampling the same biofilm, which may have inflated the reported statistical confidence, and in the other, no replicates were sampled. The 52-h adjustment period after changing the potential likely decreased the magnitude of the transcriptional response, but it was chosen to facilitate comparison to proteomics data (19). On a shorter time scale, transcription of genes for upregulated pathways might experience a burst, as the cells adjust to the new regulatory regimen, but would relax back to the new steady state once the proper balance of proteins has been reached (see, e.g., reference 34). This also allowed us to be certain that there were no lingering effects from running CV and that any changes that were detected in the transcriptome represented a steady-state adjustment and not a transient spike due to short-term stress. Also, the fundamental metabolism of “*Ca.* Tenderia electrophaga” does not change. Electrons are still being transferred from the cathode to the ETC, and CO_2_ fixation is still the sole carbon source.

Our results suggest that “*Ca.* Tenderia electrophaga” is primarily responsible for EET linked to CO_2_ fixation in biocathode-MCL biofilms. The dominance of “*Ca.* Tenderia electrophaga” transcriptional activity and its correlation to current density suggest that it can account for the majority of the current. Further substantiating this hypothesis are two recent reports of unclassified bacteria, thought to be within the *Chromatiales*, which were dominant members of freshwater biocathode communities (17, 18). While *Marinobacter* spp. are capable of cathode oxidation (21, 22, 35), isolates of *Marinobacter* sp. and *Labrenzia* sp. from the MCL community are capable of only weak current production at the biocathode or iron oxidation in monoculture (13).

On the basis of a prior analysis of biocathode-MCL performed using slow-scan-rate CV (19), we predicted the current to be approximately halved when the electrode potential was switched from 310 mV to 470 mV. However, as previously observed (19), for biocathode-MCL, switching to and maintaining a more positive potential (470 mV) over a much longer time period (>50 h) resulted in an increase in current attributed to O_2_ reduction after the initial adjustment period. This increase in the magnitude of the current is interpreted as the result of the need to make up for the decrease in energy available per electron at the higher potential. Employing a derivation of the Nernst equation (ΔG = −nFΔE^°′^) to estimate the theoretical yield of O_2_ reduction to H_2_O using an electron donor at a potential of 310 mV yields −47 kJ/mol e^−^, indicating the requirement of an additional ~61 kJ/mol e^−^ to reduce NAD(P)^+^. Assuming that the energy required to pump protons across the membrane is ~21 kJ/mol, “*Ca.* Tenderia electrophaga” could export two H^+^ per e^−^ at the optimal potential and would need to use three H^+^ to reduce NAD(P)^+^. This results in a theoretical balance for electron utilization by the forward versus reverse electron transport pathways of ~60%/40% to produce NAD(P)H. More electrons would need to go to the forward path to generate a proton gradient for ATP production. In comparison, using an electron donor at a potential of 470 mV reduces the ΔG to −32 kJ/mol e^−^, lowering the theoretical yield to ~1.5 H^+^ exported per e^−^, and the larger ΔE°′ between the electrode and NAD(P)H requires at least four H^+^ translocated per NAD(P)^+^ reduced. This results in an electron utilization balance of at least 80% and 20% for the forward and reverse pathways, respectively. Thus, theoretically, at least twice as many electrons are needed to generate the same amount of reducing equivalents for CO_2_ fixation at the more positive potential. During growth under standard culture conditions, the acidophilic iron oxidizer *A. ferrooxidans* is predicted to have a ratio closer to 90/10% for the forward versus reverse pathways, including the proton gradient necessary to generate ATP (36). Recent experimental evidence suggests that the ratio of electron utilization by the forward and reverse pathways during growth on an electrode is close to 15:1 in *A. ferrooxidans* (15).

**FIG 5:**
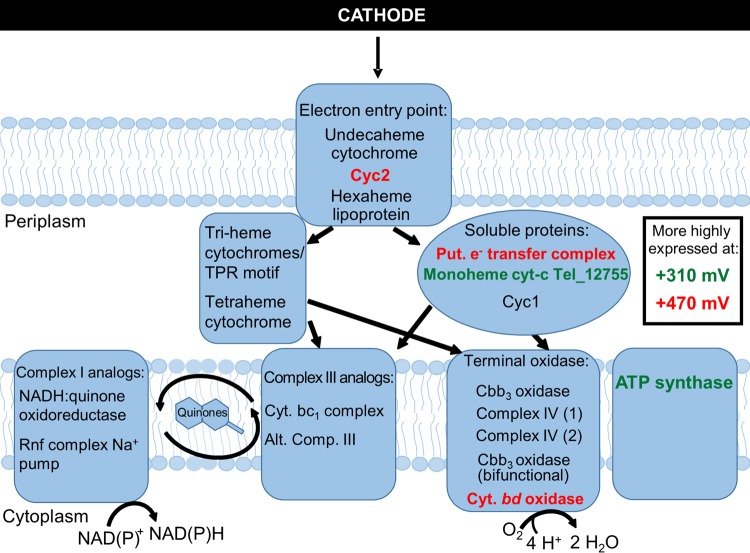
Schematic of potential EET/ETC routes in “*Ca.* Tenderia electrophaga.” Genes or complexes labeled in red were more highly expressed at 470 mV; those in green were more highly expressed at 310 mV. TPR, tetratricopeptide repeat.

Redundancy of electron transport chain components suggests metabolic flexibility in “*Ca.* Tenderia electrophaga” dependent upon the potentials of electron donors and the local redox environment (Fig. 5). Thermodynamics dictates that the balance of electrons passing through the two branches of the ETC depends upon the electrode potential. One possible method of controlling this ratio would be modulating the abundance of the periplasmic links between EET and the ETC. Several proteins could potentially make up this link, but their roles are presently unclear. One highly differentially expressed gene (Tel_12755) encodes a soluble monoheme cyt-*c* that could fulfill this role. Hierarchical clustering of potential electron transfer components by expression led to identification of several distinct clusters (Fig. 3). One cluster contains the ACIII and NUOR genes, which suggests that these complexes may be functionally linked. The canonical cytochrome cbb_3_ oxidase is part of a cluster with succinate dehydrogenase (complex II) and the cytochrome *bc*_1_ complex. This may indicate that they form part of a forward ETC, which would enable the use of stored reserves in the form of glycogen.

Among the proteins possibly involved in ET at the outer membrane, the Cyc2 homolog encoded by Tel_03480 was more highly expressed at 470 mV, consistent with the lower energetic yield per electron. This protein has previously been implicated in EET in the acidophilic iron-oxidizing bacterium *A. ferrooxidans* ATCC 19859 (31, 37, 38). The E_M_ of Cyc2 in *A. ferrooxidans* is reported to be 560 mV at pH 4.8 (27). This is within the range that might be expected for a protein accepting electrons from a cathode at the potentials tested, although the midpoint potential of Cyc2 in “*Ca.* Tenderia electrophaga” is likely different. This strongly suggests that Cyc2 is involved in EET with the electrode in “*Ca.* Tenderia electrophaga,” although its exact role given the single heme and lack of transmembrane helices remains unknown.

The majority of CBB cycle genes and pentose phosphate cycle genes appear to be more highly expressed at 310 mV. This likely reflects a higher rate of CO_2_ fixation at 310 mV than at 470 mV. Furthermore, the higher relative expression of the form IAc RuBisCO genes and their associated carboxysomes at 470 mV may reflect the need to increase the efficiency of CO_2_ fixation due to the lower energy availability. However, even at 470 mV, the overall transcript abundance of form IAq is still twice as high as that of form IAc. Metatranscriptomics of biocathode MCL supports previous work by our group that identified “*Ca.* Tenderia electrophaga” as the primary electroautotroph. It appears to be the key member of the community, and the notion of its predicted lifestyle as an electroautotroph is supported by several key findings. “*Ca.* Tenderia electrophaga” was more active than any other constituent, and its activity was positively correlated with current density. Genes for proteins previously predicted to be involved in EET in “*Ca.* Tenderia electrophaga,” including Cyc2, were more highly expressed at 470 mV, when more electrons are needed to generate the same amount of PMF. The membrane-bound ETC that is necessary to generate the ATP and reducing power for CO_2_ fixation was active at both potentials, but a potential complex IV analog and several that may be involved in the “downhill” branch of the ETC showed increased expression in response to changes in electrode potential. These changes were consistent with the need to use more electrons to obtain the same amount of energy. Most pathways of central carbon metabolism were more highly expressed at 310 mV, though not at the individual gene level, suggesting a higher metabolic rate. The evidence for involvement of specific proteins or complexes in EET and ETC is correlative in nature but reduces the list of potential targets for the future confirmatory studies that are in progress. Further investigations with better temporal resolution may yield additional insights into the dynamics of the response to the changes in potential. Heterologous expression and biochemical measurements of predicted components of EET and the ETC may help identify functional roles. This work provides insight into the mechanisms for electroautotrophic growth in this biocathode community that will assist in engineering biocathode communities.

## MATERIALS AND METHODS

### Biofilm growth and sampling.

Samples for RNA-seq were grown in artificial seawater medium (ASW) as previously described (13). Inocula consisted of cells detached by vortex mixing and sonication from a 3-cm-by-3-cm portion of a carbon cloth electrode and were counted using an Accuri C6 flow cytometer (BD Bioscience, San Jose, CA) as described previously (19). Sterile 2-liter dual-chambered microbial fuel cell reactors (Adams and Chittenden Scientific Glass) containing a graphite coupon (3 cm by 9 cm by 0.2 cm) were inoculated with standardized inocula estimated to contain 2 × 10^5^ cells.

Four reactors were inoculated with the same inoculum, and cells were grown under standard conditions (310 mV SHE) until the maximum current (12 to 65 mA·m^−2^) was reached. At this point, CV was recorded from 610 mV to 260 mV and back to 610 mV at a scan rate of 0.2 mV/s. After CV, two reactors remained poised at 310 mV, and two were shifted to a suboptimal potential (470 mV SHE) where CV indicates that the rate of electron uptake is approximately half of that at the “optimal” potential. After 52 h, the biofilm was scraped from both sides of the cathode with a sterile razor blade, immediately immersed in RNAlater (Life Technologies, Grand Island, NY), and stored at 4°C overnight prior to freezing at −20°C until total RNA extraction was performed. This experiment was repeated 7 days later with a new inoculum. This gave two pairs of biological replicates for each treatment and for each inoculum, which helps to control for variability caused by small differences in the community present in each inoculum.

### RNA extraction, library preparation, and sequencing.

Biofilm RNA was extracted using an established protocol (39) with the following modification: instead of 0.8 g of 0.5-mm-diameter glass beads, 250 µl of zirconia beads (Life Technologies, Inc.) was used. The extracted RNA samples were subjected to DNase treatment using Turbo DNase (Life Technologies, Inc.) and were cleaned up using RNA Clean & Concentrator-5 (Zymo Research). The DNase treatment was repeated once for each sample to ensure the removal of contaminating DNA. Ribosomal RNA was depleted from RNA samples using a Ribo-Zero rRNA Removal kit for Bacteria (Illumina, San Diego, CA). RNA library preparation was carried out using a NEBNext Ultra RNA Library Prep kit for Illumina (New England Biolabs, Ipswich, MA) according to the manufacturer’s recommended protocol. The sequence reads of RNA libraries were acquired on a MiSeq instrument under automated software control (version 2.2.0; Illumina, San Diego, CA). Raw reads that passed the initial quality filter step were submitted to the NCBI Sequence Read Archive (http://www.ncbi.nlm.nih.gov/bioproject).

### Metatranscriptome alignment and statistical analysis of gene expression.

Metatranscriptome reads were trimmed for quality using Trimmomatic 0.30 with a sliding window set to 5 bases with a minimum score of 25, and the first 8 bases of the sequence and any leading bases with a quality score less than 20 were cropped (40). Individual reads with less than 50 bases remaining were discarded. A biocathode-MCL metagenomic assembly, available at http://www.ncbi.nlm.nih.gov (A. P. Malanoski, B. Lin, Z. Wang, B. J. Eddie, W. J. Hervey IV, and S. M. Strycharz-Glaven, unpublished data), was modified by removing contigs from the bins corresponding to PacBio sequenced genomes of “*Ca.* Tenderia electrophaga,” *Labrenzia* sp. CP4, and *Marinobacter* sp. CP1 (CP013099.1, CP013100.1, CP011929.1, CP011927.1, and CP011928.1) and contigs that were >98% identical to these genomes over >90% of their length. The modified metagenome sequence is available at https://figshare.com/articles/Biocathode_MCL_Curated_Metagenome/4282631. Bowtie 2 (41) was used to align trimmed paired-end reads and remaining singleton reads to the modified metagenome using the following options: -D 30 -R 3 -N 1 -L 15-i S,1,0.50—score-min L,0,−0.15.

The resulting SAM files were converted to sorted BAM files with SAMtools version 1.1, and read counts per contig were calculated using the idxstats function (42). The relative activity of each genome or metagenomic bin was calculated as the sum of RNA-seq reads mapping to the genome or bin divided by the length of the genome or bin in nucleotides as a percentage of activity in the library. Read counts per gene were calculated with the featureCount tool in the subread package (43). EdgeR (44, 45) was used to normalize read counts by the trimmed mean of M-values method and to determine the statistical significance of differences in read counts for each annotated gene and pseudogene in “*Ca.* Tenderia electrophaga” using the likelihood-ratio test. Benjamini-Hochberg adjustment was used to reduce the rate of false discovery due to multiple-hypothesis testing (46). A multifactor experimental design was used to separate changes in expression likely to be due to the use of the different inocula from changes in expression resulting from the change in electrode potential (47). Genes with an FDR of <0.05 for differential expression were considered differentially expressed. Values reported for gene expression under each set of conditions correspond to the log_2_ value of the number of reads per gene after normalization (48) (see Table S3 in the supplemental material). To facilitate analysis of differences between genes in relative expression levels, overall gene expression was calculated as log counts per minute (LogCPM) minus the log_2_ of the length of the gene divided by 1,000, roughly equivalent to the commonly used parameter of reads per kilobase per million (49). For clustering genes by change in expression, gene expression was centered at 0 for each inoculum. Euclidean distance matrices were calculated, and genes were clustered using the Ward method implemented in R package “pheatmap” version 0.7.7 (50).

10.1128/mSystems.00002-17.5 TABLE S3  Expression of all annotated “*Ca.* Tenderia electrophaga” genes. Data in columns represent a combination of gene annotation, EdgeR output, and calculated relative change in expression. Column heading abbreviations: logFC, log_2_-fold change in expression from 310 mV to 470 mV SHE (genes with a positive value were more highly expressed at 470 mV, and those with a negative value were more highly expressed at 310 mV); FDR, false-discovery rate; CPM, normalized log_2_ counts per million. Coloring in logFC column is as follows: green, value > 0.5; red, value < 0.5. Coloring in FDR column is as follows: green, value < 0.01; yellow, value < 0.02; red, value < 0.05. Coloring of data (ΔlogCPM) in inoculum columns represents a linear scale, with blue indicating low values and yellow indicating high values. Download TABLE S3, XLSX file, 1.3 MB. Copyright © 2017 Eddie et al. 2017 Eddie et al. This content is distributed under the terms of the Creative Commons Attribution 4.0 International license.

Correlations between current density and the relative abundance of mRNA from each metagenomic genome or bin were calculated as Pearson’s product-moment correlation coefficients (51). The critical value for a significant correlation for a two-tailed test with a sample size of *n =* 4 is 0.9 for a *P* value of 0.1. A higher *P* value cutoff for the correlation is justified because this is a different kind of statistical test and it assumes a linear relationship between the data, which is likely not the case for most genes. Furthermore, there was a large variability between samples collected under the same conditions (current magnitude, increasing/decreasing current, and community composition) that would prevent the nearly perfect correlation that a *P* value of 0.05 would require.

### Accession number(s).

All sequences produced in this study are available at http://www.ncbi.nlm.nih.gov/bioproject under BioProject number PRJNA244670. Raw reads that passed the initial quality filter step were submitted to the NCBI Sequence Read Archive (http://www.ncbi.nlm.nih.gov/sra) under SRP043535 with the sample identifiers SAMN03462067 to SAMN03462074.

## References

[1] Tremblay PL, Zhang T 2015 Electrifying microbes for the production of chemicals. Front Microbiol 6:201. doi:10.3389/fmicb.2015.00201. 25814988 PMC4356085

[2] Rabaey K, Rozendal RA 2010 Microbial electrosynthesis—revisiting the electrical route for microbial production. Nat Rev Microbiol 8:706–716. doi:10.1038/nrmicro2422. 20844557

[3] Lovley DR, Nevin KP 2013 Electrobiocommodities: powering microbial production of fuels and commodity chemicals from carbon dioxide with electricity. Curr Opin Biotechnol 24:385–390. doi:10.1016/j.copbio.2013.02.012. 23465755

[4] Lohner ST, Deutzmann JS, Logan BE, Leigh J, Spormann AM 2014 Hydrogenase-independent uptake and metabolism of electrons by the archaeon *Methanococcus maripaludis*. ISME J 8:1673–1681. doi:10.1038/ismej.2014.82. 24844759 PMC4817615

[5] Ross DE, Flynn JM, Baron DB, Gralnick JA, Bond DR 2011 Towards electrosynthesis in *Shewanella*: energetics of reversing the *mtr* pathway for reductive metabolism. PLoS One 6:e16649. doi:10.1371/journal.pone.0016649. 21311751 PMC3032769

[6] Bose A, Gardel EJ, Vidoudez C, Parra EA, Girguis PR 2014 Electron uptake by iron-oxidizing phototrophic bacteria. Nat Commun 5:3391. doi:10.1038/ncomms4391. 24569675

[7] Deutzmann JS, Sahin M, Spormann AM 2015 Extracellular enzymes facilitate electron uptake in biocorrosion and bioelectrosynthesis. mBio 6:e00496-15. doi:10.1128/mBio.00496-15. 25900658 PMC4453541

[8] Malik S, Drott E, Grisdela P, Lee J, Lee C, Lowy DA, Gray S, Tender LM 2009 A self-assembling self-repairing microbial photoelectrochemical solar cell. Energy Environ Sci 2:292–298. doi:10.1039/B816417G.

[9] Strycharz-Glaven SM, Glaven RH, Wang Z, Zhou J, Vora GJ, Tender LM 2013 Electrochemical investigation of a microbial solar cell reveals a nonphotosynthetic biocathode catalyst. Appl Environ Microbiol 79:3933–3942. doi:10.1128/aem.00431-13. 23603672 PMC3697567

[10] Yates MD, Eddie BJ, Kotloski NJ, Lebedev N, Malanoski AP, Lin BC, Strycharz-Glaven SM, Tender LM 2016 Toward understanding long-distance extracellular electron transport in an electroautotrophic microbial community. Energy Environ Sci 9:3544–3558. doi:10.1039/c6ee02106a.

[11] Strycharz SM, Malanoski AP, Snider RM, Yi H, Lovley DR, Tender LM 2011 Application of cyclic voltammetry to investigate enhanced catalytic current generation by biofilm-modified anodes of *Geobacter sulfurreducens* strain DL1 vs. variant strain KN400. Energy Environ Sci 4:896–913. doi:10.1039/C0EE00260G.

[12] Torres CI, Marcus AK, Lee HS, Parameswaran P, Krajmalnik-Brown R, Rittmann BE 2010 A kinetic perspective on extracellular electron transfer by anode-respiring bacteria. FEMS Microbiol Rev 34:3–17. doi:10.1111/j.1574-6976.2009.00191.x. 19895647

[13] Wang Z, Leary DH, Malanoski AP, Li RW, Hervey WJ, Eddie BJ, Tender GS, Yanosky SG, Vora GJ, Tender LM, Lin B, Strycharz-Glaven SM 2015 A previously uncharacterized, nonphotosynthetic member of the *Chromatiaceae* is the primary CO_2_-fixing constituent in a self-regenerating biocathode. Appl Environ Microbiol 81:699–712. doi:10.1128/AEM.02947-14. 25398855 PMC4277585

[14] Summers ZM, Gralnick JA, Bond DR 2013 Cultivation of an obligate Fe(II)-oxidizing lithoautotrophic bacterium using electrodes. mBio 4:e00420-12. doi:10.1128/mBio.00420-12. PMC3560526 23362318

[15] Ishii T, Kawaichi S, Nakagawa H, Hashimoto K, Nakamura R 2015 From chemolithoautotrophs to electrolithoautotrophs: CO_2_ fixation by Fe(II)-oxidizing bacteria coupled with direct uptake of electrons from solid electron sources. Front Microbiol 6:994. doi:10.3389/fmicb.2015.00994. 26500609 PMC4593280

[16] Nevin KP, Hensley SA, Franks AE, Summers ZM, Ou J, Woodard TL, Snoeyenbos-West OL, Lovley DR 2011 Electrosynthesis of organic compounds from carbon dioxide is catalyzed by a diversity of acetogenic microorganisms. Appl Environ Microbiol 77:2882–2886. doi:10.1128/AEM.02642-10. 21378039 PMC3126412

[17] Rothballer M, Picot M, Sieper T, Arends JB, Schmid M, Hartmann A, Boon N, Buisman CJN, Barrière F, Strik DP 2015 A monophyletic group of unclassified γ-proteobacteria dominates in a mixed culture biofilm of high-performing oxygen reducing biocathode. Bioelectrochemistry 106(Part A):167–176. doi:10.1016/j.bioelechem.2015.04.004. 25912513

[18] Desmond-Le Quéméner E, Rimboud M, Bridier A, Madigou C, Erable B, Bergel A, Bouchez T 2016 Biocathodes reducing oxygen at high potential select biofilms dominated by *Ectothiorhodospiraceae* populations harboring a specific association of genes. Bioresour Technol 214:55–62. doi:10.1016/j.biortech.2016.04.087. 27126080

[19] Leary DH, Hervey WJ, Malanoski AP, Wang Z, Eddie BJ, Tender GS, Vora GJ, Tender LM, Lin B, Strycharz-Glaven SM 2015 Metaproteomic evidence of changes in protein expression following a change in electrode potential in a robust biocathode microbiome. Proteomics 15:3486–3496. doi:10.1002/pmic.201400585. 26260905

[20] Eddie BJ, Wang Z, Malanoski AP, Hall RJ, Oh SD, Heiner C, Lin B, Strycharz-Glaven SM 2016 ‘*Candidatus* Tenderia electrophaga’, an uncultivated electroautotroph from a biocathode enrichment. Int J Syst Evol Microbiol 66:2178–2185. doi:10.1099/ijsem.0.001006. 26957484

[21] Debuy S, Pecastaings S, Bergel A, Erable B 2015 Oxygen-reducing biocathodes designed with pure cultures of microbial strains isolated from seawater biofilms. Int Biodeterior Biodegrad 103:16–22. doi:10.1016/j.ibiod.2015.03.028.

[22] Rowe AR, Chellamuthu P, Lam B, Okamoto A, Nealson KH 2014 Marine sediments microbes capable of electrode oxidation as a surrogate for lithotrophic insoluble substrate metabolism. Front Microbiol 5:784. doi:10.3389/fmicb.2014.00784. 25642220 PMC4294203

[23] Barco RA, Emerson D, Sylvan JB, Orcutt BN, Jacobson Meyers ME, Ramírez GA, Zhong JD, Edwards KJ 2015 New insight into microbial iron oxidation as revealed by the proteomic profile of an obligate iron-oxidizing chemolithoautotroph. Appl Environ Microbiol 81:5927–5937. doi:10.1128/AEM.01374-15. 26092463 PMC4551237

[24] Nagalakshmi U, Wang Z, Waern K, Shou C, Raha D, Gerstein M, Snyder M 2008 The transcriptional landscape of the yeast genome defined by RNA sequencing. Science 320:1344–1349. doi:10.1126/science.1158441. 18451266 PMC2951732

[25] Pokkuluri PR, Londer YY, Duke NE, Long WC, Schiffer M 2004 Family of cytochrome *c* 7-type proteins from *Geobacter sulfurreducens*: structure of one cytochrome *c* 7 at 1.45 Å resolution. Biochemistry 43:849–859. doi:10.1021/bi0301439. 14744127

[26] Bird LJ, Bonnefoy V, Newman DK 2011 Bioenergetic challenges of microbial iron metabolisms. Trends Microbiol 19:330–340. doi:10.1016/j.tim.2011.05.001. 21664821

[27] Castelle C, Guiral M, Malarte G, Ledgham F, Leroy G, Brugna M, Giudici-Orticoni MT 2008 A new iron-oxidizing/O_2_-reducing supercomplex spanning both inner and outer membranes, isolated from the extreme acidophile *Acidithiobacillus ferrooxidans*. J Biol Chem 283:25803–25811. doi:10.1074/jbc.M802496200. 18632666 PMC3258861

[28] Mitchell P 1961 Coupling of phosphorylation to electron and hydrogen transfer by a chemi-osmotic type of mechanism. Nature 191:144–148. doi:10.1038/191144a0. 13771349

[29] Levar CE, Hoffman CL, Dunshee AJ, Toner BM, Bond DR 2017 Redox potential as a master variable controlling pathways of metal reduction by *Geobacter sulfurreducens*. ISME J 11:741–752. doi:10.1038/ismej.2016.146. 28045456 PMC5322298

[30] Zacharoff L, Chan CH, Bond DR 2016 Reduction of low potential electron acceptors requires the CbcL inner membrane cytochrome of *Geobacter sulfurreducens*. Bioelectrochemistry 107:7–13. doi:10.1016/j.bioelechem.2015.08.003. 26407054

[31] Yarzábal A, Brasseur G, Ratouchniak J, Lund K, Lemesle-Meunier D, DeMoss JA, Bonnefoy V 2002 The high-molecular-weight cytochrome *c* Cyc2 of *Acidithiobacillus ferrooxidan*s is an outer membrane protein. J Bacteriol 184:313–317. doi:10.1128/JB.184.1.313-317.2002. 11741873 PMC134758

[32] Ishii S, Suzuki S, Norden-Krichmar TM, Tenney A, Chain PS, Scholz MB, Nealson KH, Bretschger O 2013 A novel metatranscriptomic approach to identify gene expression dynamics during extracellular electron transfer. Nat Commun 4:1601. doi:10.1038/ncomms2615. 23511466

[33] Marshall C, Ross D, Handley K, Weisenhorn P, Edirisinghe J, Henry C, Gilbert J, May H, Norman RS 2016 Metabolic reconstruction and modeling microbial electrosynthesis. bioRxiv doi:10.1101/059410. PMC5566340 28827682

[34] Tomasch J, Gohl R, Bunk B, Diez MS, Wagner-Döbler I 2011 Transcriptional response of the photoheterotrophic marine bacterium *Dinoroseobacter shibae* to changing light regimes. ISME J 5:1957–1968. doi:10.1038/ismej.2011.68. 21654848 PMC3223308

[35] Vandecandelaere I, Nercessian O, Faimali M, Segaert E, Mollica A, Achouak W, De Vos P, Vandamme P 2010 Bacterial diversity of the cultivable fraction of a marine electroactive biofilm. Bioelectrochemistry 78:62–66. doi:10.1016/j.bioelechem.2009.07.004. 19666244

[36] Roger M, Castelle C, Guiral M, Infossi P, Lojou E, Giudici-Orticoni MT, Ilbert M 2012 Mineral respiration under extreme acidic conditions: from a supramolecular organization to a molecular adaptation in *Acidithiobacillus ferrooxidans*. Biochem Soc Trans 40:1324–1329. doi:10.1042/BST20120141. 23176476

[37] Liu W, Lin J, Pang X, Mi S, Cui S, Lin J 2013 Increases of ferrous iron oxidation activity and arsenic stressed cell growth by overexpression of Cyc2 in *Acidithiobacillus ferrooxidans* ATCC19859. Biotechnol Appl Biochem 60:623–628. doi:10.1002/bab.1110. 23980744

[38] Jeans C, Singer SW, Chan CS, Verberkmoes NC, Shah M, Hettich RL, Banfield JF, Thelen MP 2008 Cytochrome 572 is a conspicuous membrane protein with iron oxidation activity purified directly from a natural acidophilic microbial community. ISME J 2:542–550. doi:10.1038/ismej.2008.17. 18463612

[39] Cury JA, Koo H 2007 Extraction and purification of total RNA from *Streptococcus mutans* biofilms. Anal Biochem 365:208–214. doi:10.1016/j.ab.2007.03.021. 17475197

[40] Bolger AM, Lohse M, Usadel B 2014 Trimmomatic: a flexible trimmer for Illumina sequence data. Bioinformatics 30:2114–2120. doi:10.1093/bioinformatics/btu170. 24695404 PMC4103590

[41] Langmead B, Salzberg SL 2012 Fast gapped-read alignment with bowtie 2. Nat Methods 9:357–359. doi:10.1038/nmeth.1923. 22388286 PMC3322381

[42] Li H, Handsaker B, Wysoker A, Fennell T, Ruan J, Homer N, Marth G, Abecasis G, Durbin R; 1000 Genome Project Data Processing Subgroup 2009 The sequence alignment/map format and SAMtools. Bioinformatics 25:2078–2079. doi:10.1093/bioinformatics/btp352. 19505943 PMC2723002

[43] Liao Y, Smyth GK, Shi W 2014 featureCounts: an efficient general purpose program for assigning sequence reads to genomic features. Bioinformatics 30:923–930. doi:10.1093/bioinformatics/btt656. 24227677

[44] Robinson MD, McCarthy DJ, Smyth GK 2010 edgeR: a Bioconductor package for differential expression analysis of digital gene expression data. Bioinformatics 26:139–140. doi:10.1093/bioinformatics/btp616. 19910308 PMC2796818

[45] R Core Team 2014 R: a language and environment for statistical computing, v3.1.0. R Foundation for Statistical Computing, Vienna, Austria http://www.R-project.org/.

[46] Benjamini Y, Hochberg Y 1995 Controlling the false discovery rate: a practical and powerful approach to multiple testing. J R Stat Soc Series B Stat Methodol 57:289–300. http://www.jstor.org/stable/2346101.

[47] McCarthy DJ, Chen Y, Smyth GK 2012 Differential expression analysis of multifactor RNA-Seq experiments with respect to biological variation. Nucleic Acids Res 40:4288–4297. doi:10.1093/nar/gks042. 22287627 PMC3378882

[48] Robinson MD, Oshlack A 2010 A scaling normalization method for differential expression analysis of RNA-seq data. Genome Biol 11:R25. doi:10.1186/gb-2010-11-3-r25. 20196867 PMC2864565

[49] Mortazavi A, Williams BA, McCue K, Schaeffer L, Wold B 2008 Mapping and quantifying mammalian transcriptomes by RNA-Seq. Nat Methods 5:621–628. doi:10.1038/nmeth.1226. 18516045 PMC13303166

[50] Kolde R 2013 pheatmap: Pretty Heatmaps, vR package version 0.7.7. http://CRAN.R-project.org/package=pheatmap.

[51] Pearson K 1895 Note on regression and inheritance in the case of two parents. Proc R Soc Lond 58:240–242. doi:10.1098/rspl.1895.0041.

[52] Sullivan MJ, Petty NK, Beatson SA 2011 Easyfig: a genome comparison visualizer. Bioinformatics 27:1009–1010. doi:10.1093/bioinformatics/btr039. 21278367 PMC3065679

