# Supplementary figures and images for: Metatranscriptomics Supports the Mechanism for Biocathode Electroautotrophy by “Candidatus Tenderia electrophaga”
Source: mSystems. 2017 Mar 28;2(2):e00002-17. doi: 10.1128/mSystems.00002-17 (PMC5371394; doi:10.1128/mSystems.00002-17)

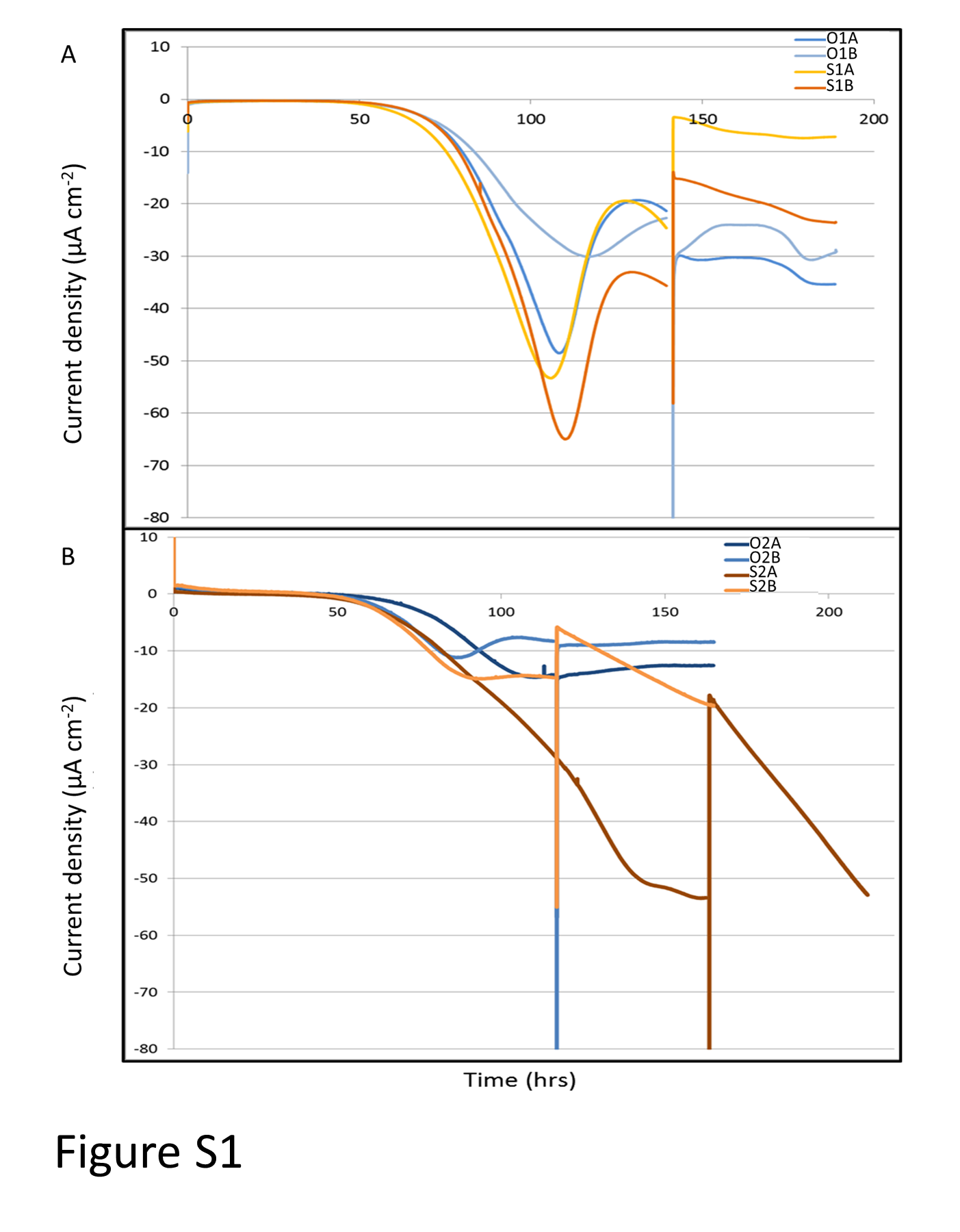

Supplement: FIG S1 [file sys002172100sf1.tif]

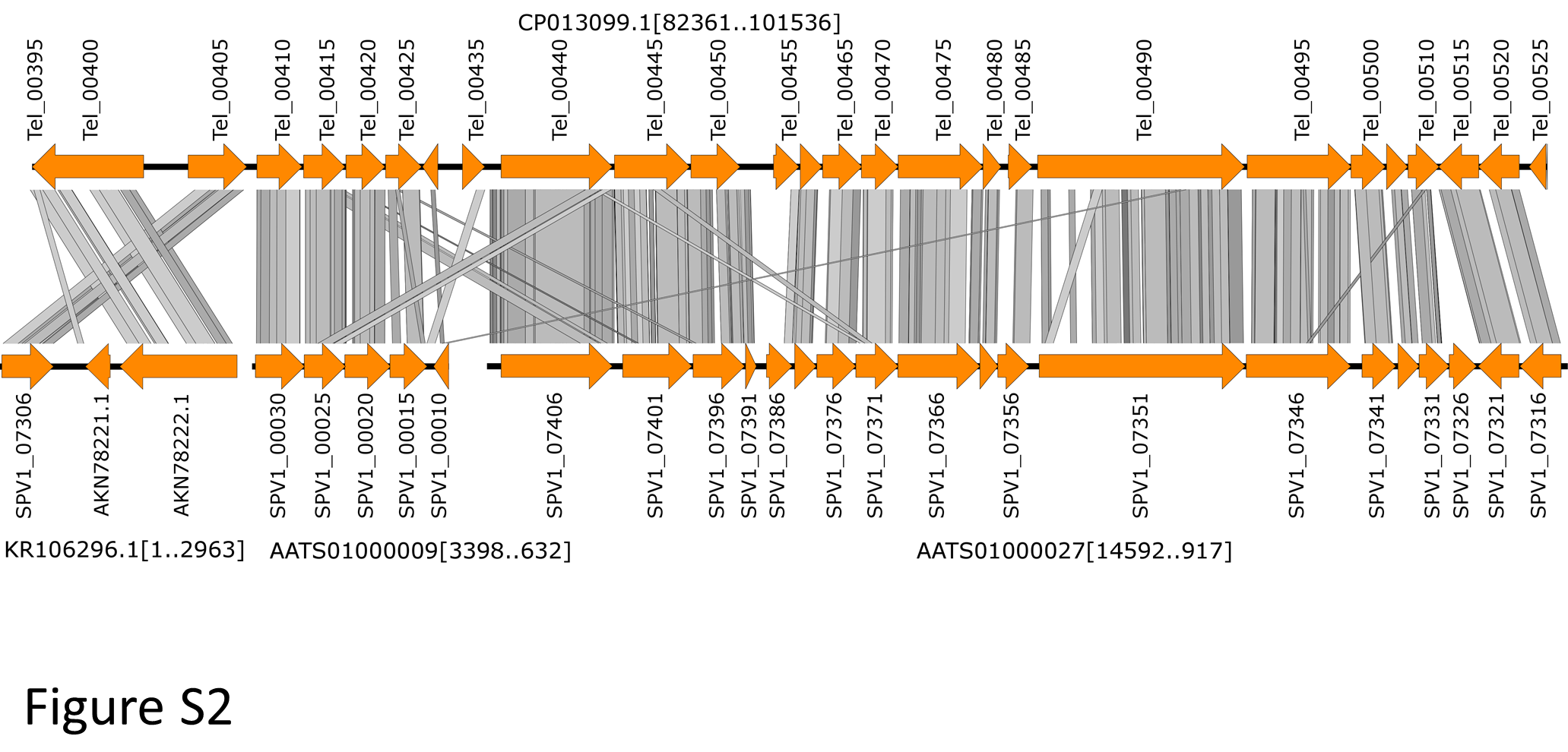

Supplement: FIG S2 [file sys002172100sf3.tif]
